# Increased Hydration Can Be Associated with Weight Loss

**DOI:** 10.3389/fnut.2016.00018

**Published:** 2016-06-10

**Authors:** Simon N. Thornton

**Affiliations:** ^1^INSERM U_1116, Université de Lorraine, Vandoeuvre les Nancy, France

**Keywords:** drinking, water, angiotensin, lipolysis, hypovolemia, hypohydration

## Abstract

This mini-review develops the hypothesis that increased hydration leads to body weight loss, mainly through a decrease in feeding, and a loss of fat, through increased lipolysis. The publications cited come from animal, mainly rodent, studies where manipulations of the central and/or the peripheral renin–angiotensin system lead to an increased drinking response and a decrease in body weight. This hypothesis derives from a broader association between chronic hypohydration (extracellular dehydration) and raised levels of the hormone angiotensin II (AngII) associated with many chronic diseases, such as obesity, diabetes, cancer, and cardiovascular disease. Proposed mechanisms to explain these effects involve an increase in metabolism due to hydration expanding cell volume. The results of these animal studies often can be applied to the humans. Human studies are consistent with this hypothesis for weight loss and for reducing the risk factors in the development of obesity and type 2 diabetes.

## Introduction

Increased water intake is associated with loss of body weight produced *via* two mechanisms, decreased feeding and increased lipolysis. The obverse also appears to be true. Mild, but chronic, hypohydration is correlated with increased body weight and its attendant dysfunctions (1). The common denominator likely is angiotensin II (AngII), the principal hormone of body fluid regulation. In what follows, this hypothesis will be tested against the available evidence (2).

AngII acts on two, seven transmembrane domain peptide receptors, AT1 and AT2. Working through the AT1 receptor AngII stimulates thirst (the act of seeking out and drinking fluids, mainly water), an appetite for sodium, the release of anti-diuretic hormone (ADH or vasopressin) to conserve water *via* the kidneys, and vasoconstriction (conserving perfusion pressure to all organs and cells). The principal physiological signal for an increase in plasma AngII is extracellular dehydration (hypovolemia) (3). The responses listed above enable the rapid return of plasma volume to normal levels, thus reducing the signal for AngII generation. This is the physiological response to hypovolemia displayed by rodents.

However, chronically elevated AngII appears to be involved in several chronic human diseases (2). Antagonists of the renin–angiotensin system (RAS) are prescribed in 85% of cases to treat cardiovascular disease (4, 5). The same antagonists are used to treat obesity (6), diabetes (7, 8), cancer (9), and Alzheimer’s disease (10). These effects could result if a subsection of the population was chronically, but mildly, hypohydrated [e.g., Ref. (11)], i.e., chronically, but mildly, hypovolemic.

These chronic diseases also involve metabolic dysfunctions (12, 13). This has been observed for cardiovascular disease (14, 15), obesity (16), diabetes (17–19), cancer (20), and Alzheimer’s disease (21). In other words, chronic hypohydration may be driving the continuous release of AngII and the metabolic dysfunction found in the chronic human diseases.

Given that in animals AngII stimulates appropriate drinking responses, why is that some humans appear not to respond appropriately to the same AngII signal? The influence of other, perhaps cognitive, factors on appropriate drinking responses has been noted in kidney stone formation, where increased water intake is recommended as a preventative measure, but compliance is difficult (22, 23). The authors noted that “not knowing the benefits of water drinking,” “not liking the taste,” and “the need to urinate frequently” influenced patient’s behavior.

## Methods

This mini-review concentrates on angiotensin and metabolic function by looking at the effect of central and peripheral manipulations of the RAS that increase drinking, reduce food intake, decrease body weight, and produce fat loss through increased lipolysis. Literature searches used keywords: angiotensin, drinking, water intake, body weight loss, obesity, diabetes, RAS antagonists, metabolism, hydration, atrial peptides, UCP1, insulin resistance, and mitochondria. Research and clinical articles are cited where there is an associated increase in water intake, a decrease in body weight, a decrease in body fat, and/or a decrease in the markers of the risk of developing obesity and type 2 diabetes. There is a large literature on the RAS and body weight regulation as well as metabolism but not all articles measured water intake and thus are not cited.

## Central AngII, Drinking, and Weight Loss

Administration of AngII into the brain of behaving animals increases drinking. Rats can consume over 2 h up to 15 ml of water in response after a single injection of AngII, depending on the dose and the site of injection (24–30). A decrease in feeding following drinking stimulated by intracranial AngII was noted early on, but this appeared to fade as the drinking response waned (31). Furthermore, in rats, chronically administered AngII over several days or weeks increased drinking (at least a doubling in daily intake), which was associated with a small decrease in food intake and a decrease in body weight, mainly through loss of fat (32–35). The decrease in body weight following the AngII infusion was greater than that in pair-fed rats.

Several mechanisms not necessarily related to the increased drinking have been suggested for this, AngII produces an increase in uncoupling protein I (33, 35). Others have suggested an increased thermic effect of food, an increased feeding hormone effect, or even an increased in stress hormone release (35). Both mechanisms imply a change in metabolic activity.

## RAS Antagonists Drinking and Weight Loss

In other rodent models of obesity, using either angiotensin-converting enzyme (ACE) inhibitors or AT1-specific antagonists increased drinking significantly with an associated decrease in food intake and body weight mainly through loss of fat. In some cases, the fat loss was specifically linked to increased lipolysis (36–41). The drinking responses ranged from a 30% increase to up to a doubling of normal intake in both rats and mice. With two AT1-specific antagonists, candesartan and losartan, this effect is observed in obese, rather than lean, rats (42, 43). Use of the renin inhibitor aliskiren in mice on both low-fat and high-fat diets demonstrated a significantly increased drinking response with a lower body weight gain and loss of body fat over a 43-day treatment period (44).

Increased drinking to RAS blockade may appear paradoxical, but it could be in response to blockade-induced increased urine flow (45, 46) or to peripheral blockade-induced increase in AI passing through the blood–brain barrier, converting to AngII in the brain, and activating hypothalamic AT1 receptors (47–49). It could also be in response to the hypovolemia produced by the RAS blockade, but no data were found to support this.

The same RAS inhibitors have been reported to be renoprotective, reduce obesity, and improve insulin sensitivity in rodents, but without recording water intakes (50–53). Similar results occurred in one human study (54), yet not in another (55), both without recorded fluid intakes. Hypohydration has been shown to lead to hyperglycemia (56), which is linked with the major problems of obesity and type 2 diabetes.

## RAS “Knockout” Mice Drinking and Weight Loss

Similar “paradoxical” results are found when the renin gene is knocked out, mice drink copiously (2.4 ± 0.1 compared with 9.2 ± 0.7 ml/day), are hyperactive, thin, have low body fat, and do not develop obesity (57). A decrease in body weight and % fat with an increase in activity was observed in renin-deficient mice on a high-fat diet, but no water intakes were given (58). Similar results occur in mice lacking the AT1 receptor (59, 60); however, no decrease in body weight was observed, despite a nearly threefold increase in drinking in these AT1-receptor KO mice (61). Furthermore, angiotensinogen-deficient mice exhibit a decrease in body weight and % body fat with an increase in activity. Water intakes were not reported in this study (62), but have been noted by others (63, 64). Similarly, in ACE gene knockout mice, water intake was doubled (from 4.2 ± 0.2 to 9.8 ± 0.5 ml/day), food intake was slightly decreased, whereas body weight and body fat were significantly decreased (fat by 10%) compared with intact controls (65).

Further details on studies on the role of the RAS in food intake and metabolic parameters are in the excellent reviews by Mathai et al. (37) and by de Kloet et al. (66). In nearly all human and animal studies, pharmacological blockade of the RAS decreases body weight, food intake, and body fat. Unfortunately, most, if not all, studies did not report measurements of water intake. This argues for clinical studies on the effects of hydration on body weight regulation.

## Mechanisms

### What Physiological Link Exists between Increased Drinking and Lipolysis?

**Figure 1:**
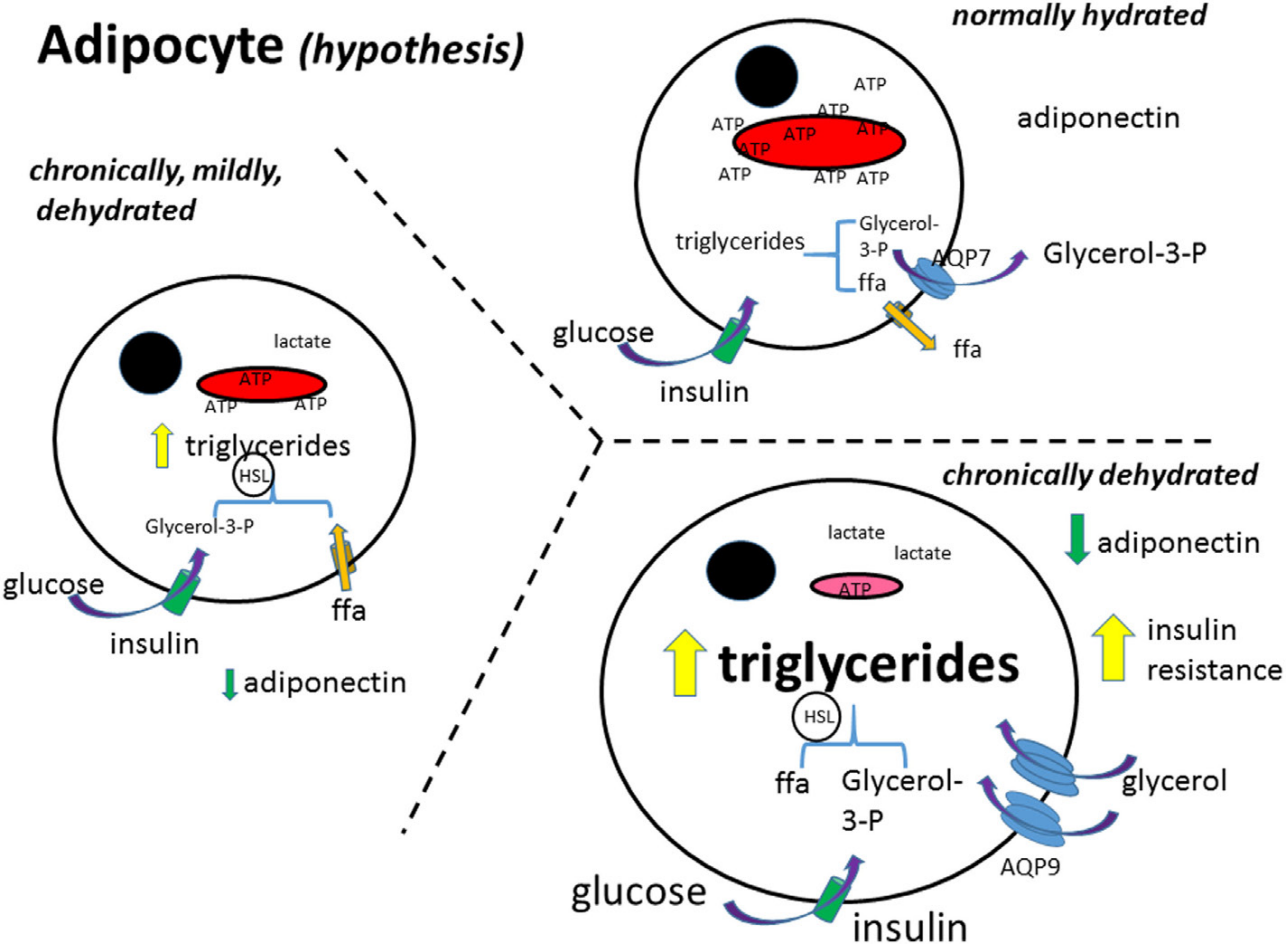
**Adipocyte metabolism (hypothesis)**. In the normally hydrated (euhydrated) adipocyte, triglycerides are formed from glucose and free fatty acid uptake, and as well broken down (lipolysis); the rate depending on the needs of the cell for ATP. Glycerol in excess is exported out of the cell. Free fatty acids (ffa) are either metabolized or exported (81). As the adipocyte gets more and more dehydrated, formation of triglycerides increases and the ffa are not able to be transformed into pyruvate and thence metabolized in the mitochondria. The glycerol transporter, aquaporin 9 (AQP9), increases, bringing in more glycerol to make more triglycerides. Glucose uptake is further stimulated by insulin, increasing also triglyceride synthesis. Black circle, cell nucleus; red structure, mitochondria; ffa, free fatty acid; aa, amino acid; AQP7 + 9, aquaporin 7 and 9; HSL, hormone-sensitive lipase.

**Figure 2:**
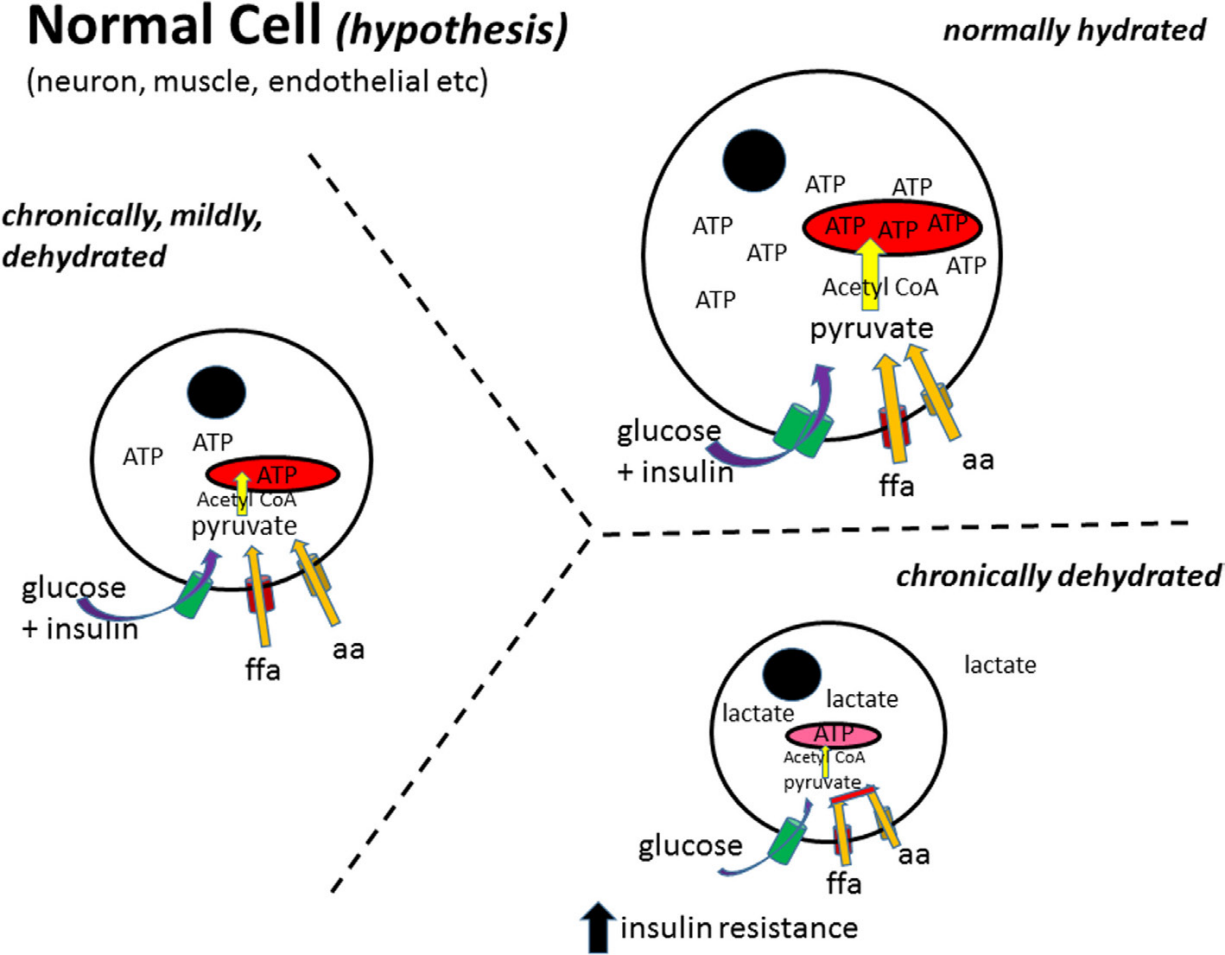
**Normal cell metabolism (hypothesis)**. In a normally hydrated (euhydrated) cell, all substrates are taken up by their appropriate transporter mechanisms and enzymatically converted to pyruvate, transported into the mitochondria, converted to acetyl-CoA, which then enters the tricarboxylic acid cycle to generate ATP (82). As the cell gets more and more dehydrated, the metabolism of free fatty acids (ffa) and amino acids (aa) to pyruvate and/or acetyl-CoA decreases producing a dependence on glucose as the main fuel source [as has been reported for obesity (16)]. Furthermore, as the cell decreases in size, the ability of insulin to stimulate glucose uptake decreases, leading to insulin resistance. Black circle, cell nucleus; red structure, mitochondria; ffa, free fatty acid; aa, amino acid.

Work in humans with administration of hypoosmotic solutions showed that there was an increase in lipolysis (67–69). The studies also show an increase in lipolysis with increased drinking indicating and, by inference, an increase in metabolism. This produced the hypothesis that increased hydration leads to an increase in cell volume and from that to increased insulin sensitivity (70–72). Furthermore, the RAS has been linked also with mitochondrial dysfunction (73–75), and treatments with RAS antagonists improved mitochondrial function (76–80). Because the same treatment induces increased water intake, this suggests that an increased hydration may enhance mitochondrial function and thus metabolism. These mechanisms are illustrated in Figures 1 and 2.

Some studies report an increase in activity with increased hydration, but the authors did not look at activity alone in the overall effects on body weight decrease.

Another plausible mechanism is that increased water intake drives thermogenesis (83–87) that would lead also to a decrease in weight gain.

Physiologically, increased water intake leads to an increase in blood volume with an attendant increase in right atrium pressure. This would release atrial natriuretic peptide (ANP), which was one of the first identified natriuretic peptides (88). This family of cardiac natriuretic peptides activates uncoupling protein 1 (UCP1) that increases fat metabolism and leads to a loss of body weight (89–95). A significant increase in UCP1 was observed in renin knockout mice fed a high-fat diet (58), and these animals drink copious amounts of water (57). Furthermore, receptors for atrial peptides have been demonstrated in brown adipose tissue (96, 97).

Physiologically, the presence of AngII is linked almost exclusively to extracellular dehydration (or extracellular thirst). The physiological stimuli for thirst are known (3) and can be broken down to intracellular and extracellular deficits. *Intracellular dehydration* involves an increase in plasma osmolality (normal levels between 295 and 300 mosmol/kg water), leading to activation of hypothalamic osmoreceptors that stimulate drinking and the release of ADH that in turn conserves hydration by increasing renal water reabsorption. This action should return plasma osmolality to normal levels, reduce the motivation to drink, and stop the release of ADH. *Extracellular dehydration*, or a decrease in blood (plasma) volume (hypovolemia), leads to renin release from the kidney, which acts enzymatically on angiotensinogen in the blood making angiotensin I (AngI). AngI is transformed by ACE into AngII. As mentioned in Section “Introduction,” AngII stimulates the seeking out and drinking of fluids (mainly water), an appetite for sodium, the release of ADH, and vasoconstriction. These actions should return plasma volume to normal levels while reducing blood AngII levels, the motivation to drink, to eat salt (mainly sodium), and the release of ADH. Most hypohydration leads to a mixture of intracellular and extracellular stimuli that should stimulate the behavioral acts of drinking and sodium intake, as well as the release of ADH, thus allowing correct regulation of body (and cellular) hydration.

Although thirst is an effective motivation in most animal studies, it may not be a sufficient or adequate stimulus for drinking in many humans, including the ill, the elderly, and infants (98). The increased blood levels of AngII indicate that part of the human population may be chronically, but mildly, hypohydrated. As suggested earlier, chronic hypohydration is driving continuous release of AngII and, by extension, the metabolic dysfunction found in cardiovascular disease, obesity, diabetes, cancer, and Alzheimer’s disease.

## Rodent and Human Hydration

In its homozygous form, the Brattleboro rat figures prominently in studies of metabolism. This animal does not produce ADH and thus urinates copiously and consequently drinks considerably, up to 200 ml/day. These animals grow more slowly than their littermate controls with ADH for the same amount of food ingested (99–101). In the Brattleboro rat, this could be due to a significantly increased metabolism as observed in neurons when measuring fluorine 18-labeled fluorodeoxyglucose uptake with a PET scanner (102).

Human studies suggest a similar effect as an increase in water intake has been associated with a decrease in body weight in obese, overweight, and normal children, and adults (103–111). Furthermore, addition of 500 ml of water before eating breakfast or a hypocaloric meal reduces energy intake (112) or increases weight loss (113). In a recent random controlled trial, there was a significant weight loss between a group eating meals with a pre-meal water load compared with the controls without a pre-meal water load (114).

## Diets, Drinking, and Weight Loss

To take this further, in rodents, a high-protein diet is associated with weight loss (115, 116) and with increased drinking (117, 118). This increased drinking may reflect the increased urine output (119, 120) needed to excrete the added urea resulting from the additional dietary protein metabolism (121). Nevertheless, based on the evidence reviewed above, the weight loss observed while on not in a high-protein diet also could be a direct result of the increased water intake. Furthermore, an increased protein diet is also associated with an increase in size and number of functionally normal liver cell mitochondria (122, 123). This would correlate with an increase in cell size following an increase in hydration as mentioned above. Finally, weight loss produced using a hypocaloric diet induces a significant (30%) increase in water intake in both young (4 months old) and old (9 months old) female mice (124).

## Discussion and Conclusion

This brief review highlights the considerable evidence that an increase in water intake, i.e., increased hydration, leads to loss of body weight. In rodent studies, the effect is clear and consistent. At the least, this requires that measurement of water intake must be included in an experiment concerning rodents and all aspects of body weight regulation, from ingestive behavior to metabolic function. An increase in metabolism is one likely mechanism for the weight loss effect (125) because this can lead to increased mitochondrial function. In adipocytes, ramping up mitochondrial activity increases lipolysis. Human studies should also address the question of hydration with the increased use of RAS antagonists in the treatment of insulin resistance (126). Body weight regulation is a complex process, and increased water intake should be part of the measures required to reduce the overall risk factors.

As mentioned in Section “Introduction,” the effects of chronic mild hypohydration extend beyond fostering obesity. Extracellular dehydration-induced AngII, and the attendant possible mitochondrial dysfunction, may contribute not only to obesity and diabetes but also to cardiovascular disease, cancer, and Alzheimer’s disease. Furthermore, there could be other “symptoms” linking these major health problems to hypohydration such as a decrease in brain volume that is also associated with Alzheimer’s disease, obesity, and diabetes and could be (127). A simple solution for reducing these modern chronic diseases would be to increase water intake across the general population. Given that hypohydration is a chronic circumstance, the effects of increased water intake would likely appear as younger groups age, as seen in schools to ameliorate childhood obesity (107, 110) and where dehydration is an issue at the start of the day (128, 129). Hypohydration occurs in France in that water intake is less than the National Nutrition Program recommendation of at least 1.5 l/day (130). The precise amounts of additional water needed and the relative importance of the different possible pathways and mechanisms remain to be specified. The implementation of such a policy would then require a public health initiative.

A limitation of this mini-review is that it concentrates mainly on papers dealing with the hypovolemia (or hypohydration)-related hormone AngII and the stimulated water intake that has effects on body weight, lipolysis, and food intake. There are a large number of studies in both animals and humans looking at the effects of RAS antagonist treatments for reducing the risk of cardiovascular disease, obesity, diabetes, cancer, and even Alzheimer’s disease where water intake, or even thirst responses, is not reported.

## Author Contributions

The author confirms being the sole contributor of this work and approved it for publication. The author would like to thank Dr. R. Norgren for his very helpful editing suggestions following detailed discussions.

## Conflict of Interest Statement

The author declares that the research was conducted in the absence of any commercial or financial relationships that could be construed as a potential conflict of interest.
